# Suppression of Innate Immunity by the Hepatitis C Virus (HCV): Revisiting the Specificity of Host–Virus Interactive Pathways

**DOI:** 10.3390/ijms242216100

**Published:** 2023-11-08

**Authors:** Sailen Barik

**Affiliations:** EonBio, 3780 Pelham Drive, Mobile, AL 36619, USA; eonbiohelp@gmail.com

**Keywords:** RNA virus, interferon, innate immunity, nonstructural protein, hepatitis C, host–virus interaction, immune suppression, protease

## Abstract

The hepatitis C virus (HCV) is a major causative agent of hepatitis that may also lead to liver cancer and lymphomas. Chronic hepatitis C affects an estimated 2.4 million people in the USA alone. As the sole member of the genus *Hepacivirus* within the *Flaviviridae* family, HCV encodes a single-stranded positive-sense RNA genome that is translated into a single large polypeptide, which is then proteolytically processed to yield the individual viral proteins, all of which are necessary for optimal viral infection. However, cellular innate immunity, such as type-I interferon (IFN), promptly thwarts the replication of viruses and other pathogens, which forms the basis of the use of conjugated IFN-alpha in chronic hepatitis C management. As a countermeasure, HCV suppresses this form of immunity by enlisting diverse gene products, such as HCV protease(s), whose primary role is to process the large viral polyprotein into individual proteins of specific function. The exact number of HCV immune suppressors and the specificity and molecular mechanism of their action have remained unclear. Nonetheless, the evasion of host immunity promotes HCV pathogenesis, chronic infection, and carcinogenesis. Here, the known and putative HCV-encoded suppressors of innate immunity have been reviewed and analyzed, with a predominant emphasis on the molecular mechanisms. Clinically, the knowledge should aid in rational interventions and the management of HCV infection, particularly in chronic hepatitis.

## 1. Hepatitis C Virus and Its Gene Expression

**Figure 1:**
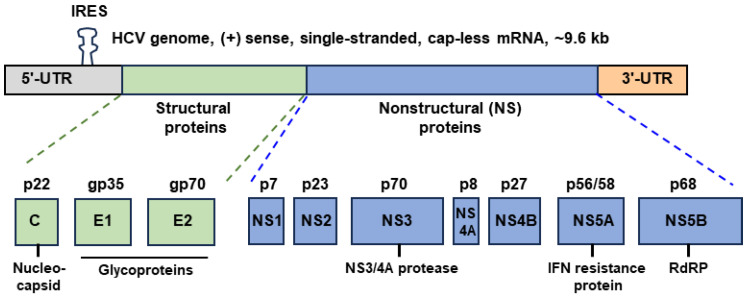
Schematic presentation of hepatitis C virus gene expression and protein products (Section 1). For each polypeptide, the alphabet names are written inside the box, and the theoretical molecular weights are on top (‘p’ = protein; ‘gp’ = glycoprotein). Thus, the nonstructural protein 1 (NS1), which is ~7 kDa in molecular weight, may be labeled NS1 as well as p7. The functions of selected proteins are described in their respective subsections in Section 3, but many proteins have multiple roles. The polypeptides, as shown in the bottom row, are the final products of programmed proteolysis of the single-precursor polypeptide, generated by genomic translation. The internal ribosome entry site (IRES) is depicted as an approximate stem–loop secondary structure. This diagram is not drawn to scale; the colors have been used simply to demarcate the different regions.

Hepatitis is most commonly caused by the namesake hepatitis viruses A, B, C, D, and E, and constitutes a major public health burden [1,2]. Though they all infect hepatic cells and affect liver function, the hepatitis viruses are diverse both genetically and clinically. Hepatitis C virus (HCV) is the only member of this group that belongs to the *Flaviviridae* family and contains a positive-sense (i.e., mRNA sense), single-stranded RNA genome of ~9.6 kb (kilobase) [3,4]. The genome is readily translated by the host cell’s translation machinery by a relatively unique initiation mechanism that is independent of the 5′-cap but utilizes an internal ribosome entry site (IRES) consisting of alternate RNA hairpin structures that are regulated by a specific microRNA, miR-122, prevalent in the liver cells. Translation of the long open-reading frame produces a single, large polyprotein, ~3000 amino acids long [5]. The polyprotein is rapidly cleaved by host and viral proteinases to generate at least 10 individual proteins [6,7,8]. The only structural proteins that comprise the enveloped virion architecture are: the nucleocapsid (also called the core, or C protein; p22) and the two envelope glycoproteins, E1 and E2. All other viral proteins are nonstructural: nonstructural 1, 2, 3, 4A, 4B, 5A, and 5B (NS1 through NS5B) (Figure 1).

These HCV proteins are not only essential for overall viral multiplication but also affect a variety of cellular functions, forming an intricate web of host–virus interactions. In this review, we will focus primarily on their involvement in the suppression of antiviral innate immunity. We begin with a brief introduction of type-I interferon (IFN), the predominant arm of innate immunity. It is to be mentioned that essentially all HCV studies use the human-hepatoma-derived HuH-7 cell line or its various derivatives, such as Huh7.5 and Huh7.5.1 [9,10,11], and the culture-adapted HCV clone, JFH1 (genotype 2a) [12,13].

## 2. Type-I Interferon

### 2.1. Interferon Induction Pathways and Factors

#### 2.1.1. The Overall Pathway

**Figure 2:**
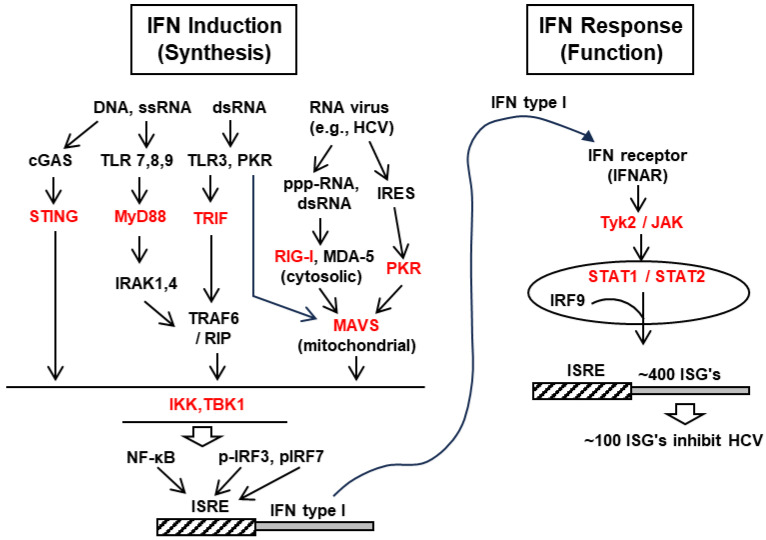
Selected members of the IFN induction and response pathways. The various abbreviations and the signaling of the pathways have been described in detail in the text (Section 2). The cascade pathways, triggered by diverse pathogenic nucleic acids (e.g., HCV RNA), and the downstream molecules are indicated. The targets of HCV (Section 3) are shown in red color.

The type-I interferon (IFN) family, represented by IFN-alpha and IFN-beta (IFN-α and -β), is a major arm of the cellular innate immunity that acts as an antiviral defense system for essentially all viruses, and HCV is no exception [14]. The IFN genes are induced in the cell upon infection by a microbe, such as viruses and bacteria, whereby their gene products are released intracellularly. Specific molecular features or patterns in these molecules, collectively termed pathogen-associated molecular patterns (PAMPs), are exemplified by viral glycoproteins and viral RNA that are double-stranded and/or contain 5′-triphosphate instead of a 5′-cap [15,16,17,18]. Each type of PAMP is sensed by a cognate cellular protein, named the pattern-recognition receptor (PRR), such as membrane-bound Toll-like receptor (TLR) or cytoplasmic RNA-binding proteins, retinoic acid-inducible gene-1 (RIG-I), or melanoma-differentiation-associated protein 5 (MDA5) [18]. Upon the binding of the PAMP, the RIG-I/MDA-5 proteins change their conformation, resulting in their recruitment to the mitochondrial adaptor protein MAVS (mitochondrial antiviral-signaling protein), also called IPS-1/Cardif/VISA (interferon-beta promoter stimulator-1/CARD-adaptor-inducing IFN-β/virus-induced signaling adaptor) [19,20,21,22,23,24]. MAVS, therefore, acts as a central innate immunity regulator, where upstream signals from several types of RNA ligands converge. The binding activates a signaling cascade that eventually leads to the site-specific phosphorylation of cytoplasmic interferon regulatory factors-3 and -7 (IRF-3/-7). The dimeric complexes of these two factors translocate to the nucleus, and along with nuclear factor kappa-B (NF-κB), bind near type-1 IFN gene promoters and promote transcriptional induction (Figure 2) [25].

#### 2.1.2. Major ‘Pattern Recognition Receptors’ (PRRs) in HCV Infection

Several lines of evidence have established RIG-I as the main PRR for HCV and which acts within minutes of virus infection [26]; RIG-I recognizes not only the 5′-triphosphate of the HCV genome, but also double-stranded regions formed by intragenomic annealing of portions of the 5′-end and the 3′-untranslated region [27,28]. The primary role of RIG-I in eliciting innate immunity to HCV is underscored by the fact that RIG-I-defective cells of the hepatocyte lineage (viz. Huh-7.5) allow robust HCV growth, and is routinely used in laboratory cell cultures.

An important molecular mechanism of RIG-I activation entails ubiquitylation, a major form of the post-translation of proteins that is ubiquitously present in living cells and is stringently regulated [29]. In ubiquitylation, which comprises a series of complex reactions, a highly conserved small protein named ubiquitin (Ubq) is covalently attached to specific target proteins, resulting in diverse changes in the properties of the target. The process comprises three sequential biochemical steps, the final one of which attaches the Ubq moiety and is catalyzed by ‘E3 ubiquitin ligases’. In the case of RIG-I, a predominant ligase is the tripartite motif-containing protein 25 (TRIM25) [30]. Subsequently, at least two other E3 ubiquitin ligases were also found to be involved in RIG-I ubiquitylation, namely, Mex3c and Riplet (also known as Reul) [31,32,33]. Thus, all three ligases are essential for RIG-I-dependent innate immunity, further attesting to the fine control of RIG-I and its strategic importance in viral immunity. Nonetheless, several other PRRs may also play a role; TLR3, for instance, possibly because it recognizes some of the same double-stranded regions that are also recognized by RIG-I [34,35,36].

#### 2.1.3. PRRs for DNA PAMPs and Their Cross-Talk with PRRs for RNA PAMPs

In mammals, double-stranded DNA is localized in the nucleus but not in the cytosol. Cytosolic DNA may occur from the loss of nuclear or mitochondrial integrity under several stress conditions, such as apoptosis or virus infection. A recently discovered cytosolic enzyme, cGAS (cyclic GMP–AMP synthase) binds to foreign DNA, liberated in the cytosol by infecting pathogens, which activates cGAS through allosteric changes. This leads to the synthesis of 2′,3′ cyclic GMP–AMP (cGAMP), a second messenger molecule that acts as an agonist of STING (stimulator of interferon genes), also known as TMEM173/MPYS/ERIS/MITA. Activated STING, residing on the endoplasmic reticulum (ER) membrane, functions downstream of MAVS and upstream of TBK1 and IKKε, eventually leading to the activation of IRF3 and NF-κB [37]. Although the cGAS–STING pathway was discovered as a DNA-activated pathway, several lines of evidence have indicated its noncanonical role in response to RNA viruses, including flaviviruses and its cross-talk with the RNA-activated pathway [38]: (i) STING directly interacts with RIG-I and MAVS [39,40]; (ii) cells or mice deficient in cGAS and/or STING support the higher growth of several RNA viruses [41,42,43]; (iii) undue mitochondrial stress may release mitochondrial DNA into the cytoplasm [44], which can then activate cGAS, and this may conceivably happen in cells infected with *Flaviviridae* viruses, such as HCV [45]. It thus stands to reason that the RNA viruses, including HCV, would also evolve ways to suppress the cGAS–STING pathway, as we will see later (Section 3).

### 2.2. Interferon Response

#### 2.2.1. The JAK–TYK2–STAT Signaling Pathway

IFN itself is not an antiviral; rather, it triggers the so-called ‘IFN response pathway’ that has been extensively reviewed [46]. In this phase of innate immunity, type-I IFN is secreted and binds to specific receptors on the target cell membrane, which activates the so-called JAK–STAT (Janus kinase/signal transduction and transcription activation) pathway [47,48], so named because the Tyr kinases JAK and TYK2 eventually phosphorylate STAT1 and STAT2 [49,50]. These two STAT proteins form a complex with IRF9 [46,51,52], and the heterotrimeric complex, named ISGF3 (IFN-stimulated gene factor 3) translocates to the nucleus, where it binds to the IFN-stimulated regulatory element (ISRE) sequences [53] in the promoters of ISGs (IFN-stimulated genes) and induces the transcription of the ISGs.

#### 2.2.2. HCV-Relevant IFN-Stimulated Genes (ISGs)

The functions of most of the ~450 ISGs remain unknown, but many possess broad antiviral properties. Comprehensive screening analyses of ISGs, using the overexpression of recombinant ISGs, as well as knockdown by RNA interference (RNAi), have established that each virus is inhibited by multiple ISGs [54,55,56,57,58,59,60]. Some of the major ISGs that inhibit HCV have been reviewed recently [61]. They include tripartite motif-containing 56 (TRIM56), viperin, and the DExD/H box helicase (DDX60), 2′,5′-oligoadenylate (2-5A) synthetase (OAS), RNA-dependent protein kinase (PKR), MxA, and proteins of the IFIT (IFN-induced proteins with tetratricopeptide repeat motifs) family, including the transmembrane IFITM proteins [62,63,64,65]. The ISGs target specific steps in the viral life cycle to inhibit the virus. IFITM1, for example, interacts with CD81 and occludin, two coreceptors of HCV, which disrupts virus entry into the host cell [65]. OAS is an enzyme that catalyzes the synthesis of 2-5A; the binding of 2-5A to latent RNase L activates the latter, which then cleaves all single-stranded cellular and viral RNA. This leads to autophagy and apoptosis of the cell [66], which abrogates virus growth. At the same time, some of the product RNA fragments induce the production of IFN-β in the proper cellular context, amplifying antiviral innate immunity [67]. Activation of the PKR catalytic function proceeds through dsRNA binding, dimerization, and autophosphorylation. PKR-mediated phosphorylation inhibits the translation factor eIF-2α, which blocks protein synthesis, and hence viral replication, as reviewed recently [68].

#### 2.2.3. HCV-Relevant IFN-Effector Genes (IEGs)

In parallel studies, genome-wide functional screening using gene-specific small interfering RNAs (siRNAs) [58,69] identified over one hundred host genes that mediated the anti-HCV effect of IFN-α in hepatocytes. In these screening procedures, the hepatoma cells were first transfected with siRNAs incubated with IFNα and then infected with HCV to identify the siRNAs that rescued virus growth. Intriguingly, a large percentage of these genes were nontranscriptionally induced, and therefore, they were not IFN-stimulated genes (ISGs), but could be designated as interferon-effector genes (IEGs). While the mechanisms of their IFN-effector role are very likely novel, the IEGs are diverse in nature and participate in cellular housekeeping functions, such as metabolism, post-translational modification, and RNA spliceosome assembly. Examples include: glucokinase regulator, histone acetyl transferase, asparagine-linked glycosyl transferase, and protein phosphatase 3 [69]. A large group of ISGs comprised the members of the U4/U6.U5 tri-snRNP, a major component of human spliceosome complex B and C, suggesting that the alternative splicing of cellular mRNA is important in the HCV–host interaction. As noted earlier [58], this may not be unique to HCV, since many known antiviral ISGs, such as many isoforms of OAS and MxA, are also generated by alternative splicing. In any case, it is currently unknown if or how HCV can suppress the ISGs, whereas there is substantial information on the HCV-mediated suppression of the classical IFN signaling factors, including the ISGs, as described below (Section 3).

To summarize Section 2, IFN induction in the HCV-infected cell occurs predominantly by the recognition of viral RNA by RIG-I, followed by activation of the RIG-I through post-translational modifications and conformational alterations, docking of the activated RIG-I to mitochondrial MAVS, and the eventual activation of transcription factors that promote IFN gene induction (Figure 2). The IFN, thus produced, acts on IFN-responsive cells to induce multiple ISGs, many of which have broad-spectrum antiviral functions. Thus, viral infection itself triggers the synthesis of antiviral ISGs of the cell as a cellular defense mechanism.

## 3. IFN Suppressor Proteins of HCV and the Mechanisms of Suppression

Currently, IFN conjugated with polyethylene glycol (PEG) (for enhanced stability in the body), known as pegylated IFN (PEG-IFN), often combined with ribavirin, which is another broad-spectrum antiviral, constitute the standard of care therapy for hepatitis C [70]. Even with this therapy, however, ~80% of HCV patients fail to clear the virus and develop a chronic hepatitis that may persist for decades and, as mentioned earlier, may lead to the serious complications of cirrhosis of the liver and resultant scarring, sometimes transitioning to liver cancer (hepatocellular carcinoma or HCC) [71]. IFN-resistant chronic HCV also presents a major hurdle in the global eradication of HCV, which affects ~2% of the human population. The incomplete efficacy of IFN is largely explained by studies over the last two decades showing that HCV infection can suppress the IFN pathways in cell cultures as well as in mouse models [72,73,74], indicating the evolution of viral counter-defense to suppress the cellular antiviral response. Naturally, much attention has been paid to the mechanisms of such suppression.

Cumulative evidence has revealed that the nonstructural proteins of HCV, perhaps with the exception of the RNA-dependent RNA polymerase (NS5B) and the NS1 (p7) glycoprotein, contribute to IFN suppression to one extent or another. In the following subsections, these IFN suppressor proteins and their diverse mechanisms of suppression are presented in detail.

### 3.1. NS3/NS4A Protease Complex

Being a protease complex, the NS3/NS4A pair of HCV (Figure 1) is primarily involved in the cleavage of the viral polyprotein at specific junction sequences; this occurs in both a cis and trans fashion, and in a highly orchestrated, apparently nonrandom, order. Due to this cardinal role of NS3/4A in the early step of viral gene expression, the biochemistry as well as structure of both proteins have been investigated in detail [75]. The cleavage of the NS3–NS4A junction in the precursor polypeptide is catalyzed by NS3 acting in the cis, leading to the liberation of NS3, following which, the NS3–NS4A complex cleaves NS4A–NS4B, NS4B–NS5A, and NS5A–NS5B junctions in the trans [6,76,77,78,79,80]. The chymotrypsin-like serine protease activity of NS3 resides in the amino-terminal one-third, which is also responsible for self-cleavage at the carboxy terminal sites of NS3 [81,82]. However, NS3 requires NS4A for optimal activity [80,83], and perhaps not unexpectedly, the two polypeptides have a natural affinity for each other to form a heterodimer, as demonstrated with the recombinant proteins [80]. Determination of the cocrystal structure of NS3 in complex with the NS3-activating domain of NS4A revealed and predicted several aspects of the function and regulation of the holoenzyme [75,84,85].

NS3/4A is by far the most dominant player in the suppression of IFN induction, as well as IFN response, documented in the infected cells in culture and in the liver, which is largely due to its ability to proteolytically cleave the relevant signaling proteins. To start with, NS3/4A targets the E3 ubiquitin ligase Riplet (Section 2.1) and inhibits the polyubiquitination of RIG-I, and inhibits the downstream function of the latter [31]. Some of the earliest studies showed that NS3/4A cleaves mitochondrial MAVS, the central docking protein in the IFN-induction pathway [21,86,87,88]. NS3/4A localizes on multiple organellar membranes, one of which is mainly involved in MAVS cleavage; this structure has been named the “Mitochondria-associated endoplasmic reticulum membrane” (MAM) [89]. In either case, MAVS is a pragmatic target for suppression, since, as described earlier (Section 2.1), RIG-I is the primary PRR for the RNA PAMPs of HCV, and the resultant signal from RIG-I is channeled through MAVS (Figure 2).

NS3/4A also cleaves TRIF, the adaptor of the TLR3 signaling cascade, thereby inhibiting the TLR3-triggered activation of IRF3 and NF-κB [90,91], and reducing the transcription of several IFN genes and proinflammatory genes, such as tumor necrosis factor-alpha (TNF-α). However, subsequent studies have shown that TRIF cleavage by NS3/4A may not be as extensive as that of MAVS [92]. In other words, MAVS cleavage is the overriding mechanism by which NS3/4A suppresses IFN induction (Figure 2). 

**Figure 3:**
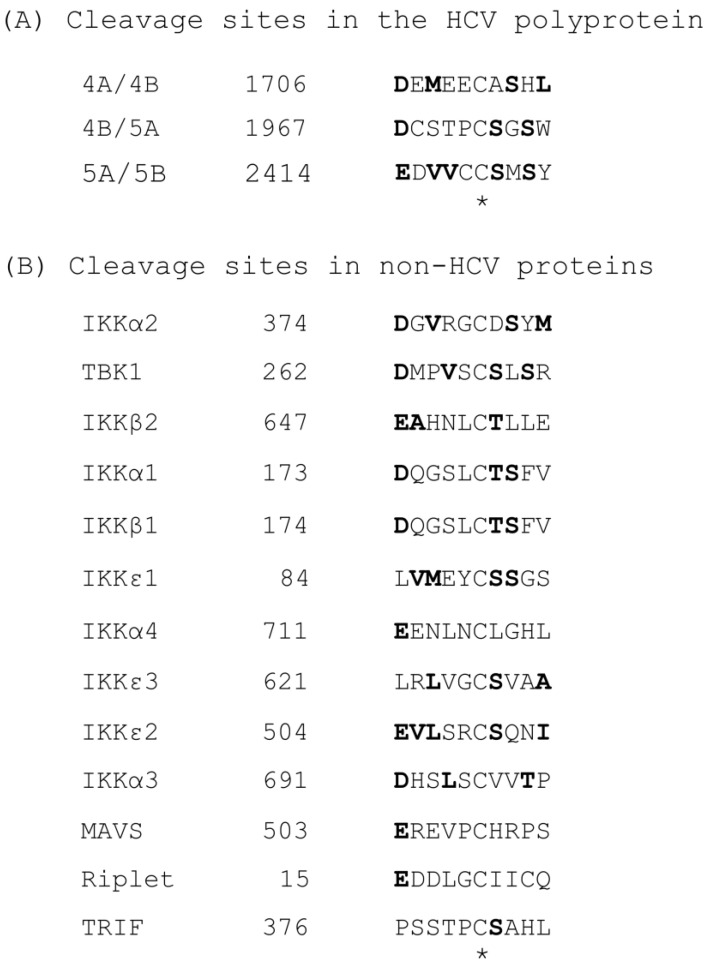
NS3/4A cleavage sequences. IKK-alpha: NP_001269; IKK-beta: NP_001547; IKK-epsilon: AAF45307. All HCV junction sequences have been described in the GenBank submission of the polyprotein sequence, CAB53095.1 [82]. Other sites are from published papers, as follows: IKK isoforms and TBK [95], MAVS [21], Riplet [32], and TRIF [90]. When there are multiple cleavage sites in a polypeptide, they are serially numbered; thus, the three sites in IKKε are denoted as IKKε1, IKKε2, and IKKε3. When at least three amino residues of conservative properties are aligned, they are shown in bold. The two asterisks mark the invariant C (Cys) residue.

The cleavage sequences, preferred by NS3/4A, have been established by several pioneering studies that are well in agreement with one another and with antiprotease drug development strategies [7,78,82,93,94]. An alignment of the cleavage sites (Figure 3, panel A) reveals a consensus of (D/E)XXXXC(A/S), where X is any amino acid residue, and the cleavage occurs after the conserved Cys (shaded). In extending our knowledge of the cleavage sequences of NS3/4A, a recent study using a recombinant NS3/NS4A complex demonstrated their ability to cleave a number of cellular proteins of the IFN pathway, namely, IKKα, IKKβ, IKKε, and TBK1 [95]. Characterization of the fragments by MALDI-TOF and LC-MS/MS (matrix-assisted laser desorption/ionization time-of-flight; liquid chromatography–triple quadrupole mass spectrometry) revealed discrete sites of cleavage in each, with significant similarity to the HCV-derived consensus, including the conserved Cys (Figure 3B). Although these results have not been pursued further, they clearly indicate a potentially larger substrate repertoire of NS3/NS4A that extends to host-cell proteins. The biological relevance of such seemingly inconsequential cleavages is not known.

### 3.2. NS2

Less studied than NS3/NS4A, NS2 also displays protease activity that cleaves TBK1/IKKε, leading to the subversion of IRF3 phosphorylation and IFN-β gene induction [96]. Like NS3/4A, NS2 also inhibits expression from MAVS-dependent promoters; however, unlike NS3/4A, NS2 does not cleave MAVS, and must inhibit its target promoters by a different mechanism [88]. It is notable that, like NS3/4A, the NS2 protein inhibits several other cellular and viral promoters, such as CCL5/RANTES, CXCL10/IP-10, and the thymidine kinase gene of herpes simplex virus, the mechanisms of which also remain unknown [88,97]. In either case, NS3/4A and NS2 are not functionally redundant, but work together and keep a tight rein on the IFN pathway.

The role of NS2 in viral polyprotein cleavage presents a novel mechanism of regulation, which we will very briefly mention here. This is primarily because NS3 can form a protease complex not only with NS4A but also with NS2. However, while NS3/4A cleaves at four downstream sites in the polyprotein, as described in Section 3.1, the NS2/3 protease mediates a single cleavage at the NS2–NS3 junction. In the NS2/NS3 protease, the catalytic triad resides in NS2, and is stimulated by the cofactor domains of NS3 that require Zn^+2^ [98,99]. Thus, in both NS3/4A and NS2/3, the protease activity requires a composite surface of domains contributed by two polypeptides, of which NS3 is common to both complexes [100,101]. However, since there is no report of any role of NS2/3 in IFN suppression, we will not discuss it any further.

### 3.3. NS4B

The nonstructural 4B (NS4B, p27) protein is overall hydrophobic, with multiple roles in HCV replication and host–virus interaction [102]. Briefly, NS4B exhibits NTPase and RNA-binding activities, regulates the RNA-dependent RNA polymerase (RdRP) activity of NS5B, affects the function of the endoplasmic reticulum, and modulates viral as well as host translation. A distinctive role of NS4B, facilitated by its amphipathic domain, is the generation of the “membranous web”, which is a novel intracellular membrane structure. This structure acts as a scaffold on which viral replication and assembly occurs. Some of these functions may cross-feed one another, such as the RNA-binding property may be important for NS4B to engage the elongating RdRP (NS5B) copying the RNA template.

Several investigators have reported the suppression of the STING–TBK1 axis (Figure 2) by NS4B [103,104,105]. NS4B directly binds STING and blocks the interaction between STING and MAVS, which is required for robust IFN-β activation. Expression of recombinant NS4B suppressed residual IFN-β activation by an NS3/4A-cleaved MAVS, suggesting that the cooperation between NS3/4A and NS4B results in a strong suppression of IFN-β induction.

### 3.4. NS5A 

A direct role of NS5A in HCV replication has not been proven, although evidence of its affinity for the 3′ ends of the HCV plus- and minus-strand RNAs has promised to shed light on this aspect [106], perhaps involving a direct interaction with the RdRP (NS5B), as was proposed for NS4B (Section 3.2). NS5A is known to assist the HCV replicative complex and virion assembly in partnership with the C protein (Section 3.4). RNA aptamers selected for their affinity to different domains of NS5A inhibit HCV RNA replication and infectious virus production in cell cultures [107]. The total interaction network of NS5A actually is much larger, as it associates with nearly two dozen host proteins involved in a multitude of cellular functions, such as apoptosis, cell-cycle control, and stress response, much of which has been detailed in a comprehensive review [108]. This best-studied property of NS5A is its facilitation of virus growth indirectly by suppressing specific signaling steps of the IFN pathways through multiple mechanisms elaborated in Section 2. Indeed, NS5A was the first HCV protein implicated in the suppression of IFN, evidenced from the investigations of patients with chronic infection by HCV of genotype 1b [109]. Since then, a large number of publications have reported various aspects of this suppression, of which a few representative ones will be discussed here in no particular order. Early studies revealed an approximately 40-amino-acid region at the center of the NS5A polypeptide that appeared to be associated with the sensitivity of HCV to the antiviral effects of IFN; this sequence was, therefore, named the “Interferon Sensitivity-Determining Region” (ISDR) [109,110,111]. Later studies showed that this role of ISDR may not be relevant in the long-term response to IFN [112,113]. 

Several researchers have shown that NS5A interferes with the TLR–MyD88 signaling axis in cultured macrophages [114], as well as in mouse liver [115], by directly binding to MyD88. Various members of the TLR family are activated by cognate ligands; thus, TLR2, TLR4, TLR7, and TLR9 are activated upon the binding, respectively, of PGN (peptidoglycan, a bacterial polymer), LPS (lipopolysaccharide of bacterial origin), R-837 (imiquimod, a synthetic imidazoquinoline amine analog to guanosine), and mCpG (mouse CpG dinucleotide). In mouse macrophage cell lines expressing recombinant NS5A, the induction of a key marker, i.e., interleukin-6 (IL-6), was inhibited. Similar inhibition was seen when the protease(s) NS3 or NS3/4A were expressed, as expected (Section 3.1), and also when NS4B was expressed. In one mechanistic study [116], NS5A was shown to inhibit IKKε, thereby reducing the phosphorylation and activation of IRF3, and, therefore, IFN-β synthesis (Figure 2). Lastly, NS5A affects a few members of the IFN response pathway. Specifically, it inhibits IFN-induced STAT1 phosphorylation and ISG synthesis [117,118], the molecular mechanism of which is unknown. NS5A also inhibits the protein kinase activity of PKR, an ISG [110,119,120]. Mutations within NS5A, including those within the ISDR (see above), can disrupt the NS5A–PKR interaction, which may contribute to the reduced IFN sensitivity of the ISDR-mutant HCV. Further studies showed that the repression of the PKR function by NS5A is mainly due to the abrogation of PKR dimerization and the resultant inhibition of its kinase activity and loss of PKR-mediated eIF-2α phosphorylation.

However, PKR–NS5A interaction is much more complex and intersects multiple other pathways [121]. Several lines of evidence point to a role of PKR as a pro-HCV agent, related to the ubiquitin-like protein ISG15, which is another interferon-stimulated gene product. In one mechanism, early in infection, ISG15 prevents the TRIM25-mediated ubiquitination of RIG-I (Section 3.1), and hence its docking to MAVS, which suppresses IFN induction. Later in the infection, the PKR-catalyzed phosphorylation of eIF2α shuts down cellular cap-dependent translation, whereas the IRES-dependent HCV translation continues largely unabated [122,123,124]. Activation of PKR may also shut down the translation of other ISGs in the IFN-treated cell. Some apparent discrepancy between different studies on the PKR–HCV interaction that used an HCV replicon-based assay ex vivo (cell culture) were likely due to different genotypes of HCV [125,126,127]. Thus, the effect of this ISG in HCV replication appears to be a balance between its pro- and antiviral functions. NS5A also physically associates with p51 and regulates the p53-dependent p21/WAF1 promoter [128], a property that is shared by several other HCV proteins, such as NS3 and core protein (Section 3.1 and Section 3.5).

A regulatory mechanism has emanated from the discovery that the post-translational phosphorylation of NS5A at specific Ser residues by multiple cellular kinases play an important role in HCV replication [129,130,131,132]. If this phosphorylation is also found to regulate the IFN suppression function of NS5A, it will certainly open multiple new directions of investigation.

### 3.5. Core Protein, C

In its role as a major structural protein of the virion, the C protein of HCV forms the viral nucleocapsid and possesses both RNA- and lipid-binding activities, allowing it to interact with genome RNA as well as the virion envelope, respectively, a hallmark of enveloped RNA viral nucleocapsids. The C protein also promotes the localization of the virus replicative complex to the lipid droplets (LDs) and cell membranes, which is essential for virion assembly and budding. In addition, the C protein brings NS5A to the LDs and for the production of infectious progeny virus particles [133,134,135,136,137]. The amphipathic and multidomain structure of the core protein in fact allows it to interact with a variety of cellular proteins and intracellular structures, which in turn influences the activity of other HCV proteins that directly suppress IFN [138,139,140,141,142]. Through such interactions with various host functions, the C protein also regulates cellular apoptosis, indirectly promoting chronic-IFN-resistant chronic HCV [139,140,141]. Several of them also lead to the suppression of a range of immune responses via direct or indirect mechanisms (Section 2). 

The C protein was shown to inhibit both the TLR- and RLR-mediated IFN response pathway in dendritic cells, which was associated with increase in unphosphorylated (inactive) STAT1 [143]. The C protein also represses IRF1 (IFN regulatory factor 1), a secondary transcription factor of the IFN system that also binds to the ISRE sequences and induces the transcription of several antiviral ISGs and immunomodulatory genes (Section 2.2), such as OAS and the interleukins IL-12 and IL-15 [144]. Repression of IRF1 by the C protein occurs at the transcriptional level and requires the STAT-1 and NF-κB consensus sequences on the IRF-1 promoter; however, the molecular structure of the protein–DNA complex remains unknown [144]. Recombinant C protein was also shown to repress the transcription of several other cellular and viral promoters, namely p53, p21/WAF1/Cip1/Sid1, c-myc, and the long terminal repeats (LTRs) of Rous sarcoma virus and human immunodeficiency virus type 1 [145,146,147]. For the p21/WAF1 promoter, the repressive activity is regulated by the protein kinase A-mediated phosphorylation of a specific Ser residue of the C protein [148]. 

In a parallel mechanism, the C protein induces expression of the suppressors of cytokine signaling (SOCS) [149], which downregulates IFN-induced STAT-1/3 phosphorylation, leading to reduced IFN signaling and the eventual loss of expression of several ISG mRNAs, such as MxA and 2′,5′-OAS [150]. Thus, the C protein in this mechanism uses the SOCS proteins to suppress the IFN response pathway to promote HCV persistence. Some of these multifaceted mechanisms of the IFN-subversive role of the C protein have been described in comprehensive review [151], which the readers may consult.

We note that there is no definitive evidence of an IFN-suppressive role of the HCV NS1 protein [152]. A few early reports of the inhibition of TLR3-dependent IFN induction by the NS1 proteins of WNV and YFV in cell culture [153,154] could not be reproduced in a more recent study [155]. The NS1 proteins of the *Flaviviridae* family are also very diverse in the primary structure, making it unlikely that the NS1 of HCV will be functionally comparable to those of WNV or YFV. Finally, there is also no report that the E glycoproteins (E1, E2) of HCV suppress IFN; therefore, these three proteins (NS1, E1, E2) (Figure 2) were not elaborated in this section.

A summary of the molecular targets of the IFN-suppressor proteins of HCV is presented below in tabular form (Table 1); a mechanistic summary is presented in Section 4.

## 4. Mechanism-Based Summary of IFN Suppression by HCV and Unanswered Questions

A review of the IFN suppression by HCV proteins, as detailed in Section 3, reveals several biochemical mechanisms that can be classified into a few representative types: 

(i) Proteolytic cleavage, such as the cleavage of many substrates by NS3/4A, and that of TBK1/IKKε by NS2. 

(ii) Inhibition of an enzyme: The kinase activity of IKKε is inhibited upon NS5A-binding [116]. 

(iii) Binding and sequestration: NS4B-binding to STING blocks STING–MAVS interaction, and NS5A sequesters MyD88. NS5A-binding to the N-terminal 488 amino acids of STAT1 also inhibits IFN-induced STAT1 phosphorylation, and hence ISG expression [127]. The C protein binds to membrane structures and proteins, acting as an adaptor between them. It also appears to have DNA-binding properties, by virtue of which it engages consensus sequences at the IRF1 promoter and represses IRF1 transcription [144].

One of the most fascinating questions to answer in the future—and perhaps also the most intractable—is how the suppressors interact with such a broad repertoire of proteins and substrates, and yet maintain substantial amounts of specificity of interaction. The repertoire often includes other viral proteins, such as the interaction of NS3 with NS4A, NS2 with NS3, and C, NS4B, and NS5A with the viral replicative complex (mainly NS5B). Related to this question is how the multiple interactions evolved over time, and in what order, while maintaining both the viral and the cellular interactions. It is conceivable that these studies will minimally require sequence information of multiple isolates of the virus, their time of appearance, the rate of mutation of the different sites, and mapping of the interacting domains. Clinically, a better knowledge of the suppressors may allow rational, structure-based drug design to inhibit them [156], which should help resolve the chronic HCV cases.

## Figures and Tables

**Table 1:** IFN-suppression proteins of HCV and their molecular targets.

HCV Protein	Target Cell Protein
NS3/NS4A	Binds to Riplet (E3 ubiquitin ligase), resulting in inhibition of RIG-I
	Cleaves MAVS
	Cleaves TRIF
NS2	Cleaves TBK1/IKKε
	Inhibits MAVS-dependent promoters; mechanism unclear
	Binds to STING; blocks STING–TBK1 interaction
NS5A	Binds to MyD88
	Inhibits STAT2 phosphorylation
	Inhibits PKR by preventing PKR dimerization
C	Increases the ratio of unphosphorylated: phosphorylated STAT1
	Induces the expression of SOCS, lowers STAT1/3 phosphorylation

Due to space constraints, only those cellular proteins that physically interact with the cognate HCV protein are shown. In other words, when downstream proteins are inhibited as a result of this direct interaction upstream, it was described earlier, but not included in this table.

## Data Availability

This article contained all the relevant data described herein.
